# MRI‐TRUS registration methodology for TRUS‐guided HDR prostate brachytherapy

**DOI:** 10.1002/acm2.13292

**Published:** 2021-07-28

**Authors:** Philip McGeachy, Elizabeth Watt, Siraj Husain, Kevin Martell, Pedro Martinez, Summit Sawhney, Kundan Thind

**Affiliations:** ^1^ Department of Medical Physics Tom Baker Cancer Centre Calgary AB Canada; ^2^ Department of Oncology University of Calgary Calgary AB Canada; ^3^ Department of Physics and Astronomy University of Calgary Calgary AB Canada; ^4^ Department of Radiation Oncology Tom Baker Cancer Centre Calgary AB Canada; ^5^ Department of Radiology and Diagnostic Imaging University of Calgary Calgary AB Canada

**Keywords:** HDR brachytherapy, MIM, MRI, prostate cancer, registration, TRUS

## Abstract

**Purpose:**

High‐dose‐rate (HDR) prostate brachytherapy is an established technique for whole‐gland treatment. For transrectal ultrasound (TRUS)‐guided HDR prostate brachytherapy, image fusion with a magnetic resonance image (MRI) can be performed to make use of its soft‐tissue contrast. The MIM treatment planning system has recently introduced image registration specifically for HDR prostate brachytherapy and has incorporated a Predictive Fusion workflow, which allows clinicians to attempt to compensate for differences in patient positioning between imaging modalities. In this study, we investigate the accuracy of the MIM algorithms for MRI‐TRUS fusion, including the Predictive Fusion workflow.

**Materials and Methods:**

A radiation oncologist contoured the prostate gland on both TRUS and MRI. Four registration methodologies to fuse the MRI and the TRUS images were considered: rigid registration (RR), contour‐based (CB) deformable registration, Predictive Fusion followed by RR (pfRR), and Predictive Fusion followed by CB deformable registration (pfCB). Registrations were compared using the mean distance to agreement and the Dice similarity coefficient for the prostate as contoured on TRUS and the registered MRI prostate contour.

**Results:**

Twenty patients treated with HDR prostate brachytherapy at our center were included in this retrospective evaluation. For the cohort, mean distance to agreement was 2.1 ± 0.8 mm, 0.60 ± 0.08 mm, 2.0 ± 0.5 mm, and 0.59 ± 0.06 mm for RR, CB, pfRR, and pfCB, respectively. Dice similarity coefficients were 0.80 ± 0.05, 0.93 ± 0.02, 0.81 ± 0.03, and 0.93 ± 0.01 for RR, CB, pfRR, and pfCB, respectively. The inclusion of the Predictive Fusion workflow did not significantly improve the quality of the registration.

**Conclusions:**

The CB deformable registration algorithm in the MIM treatment planning system yielded the best geometric registration indices. MIM offers a commercial platform allowing for easier access and integration into clinical departments with the potential to play an integral role in future focal therapy applications for prostate cancer.

**Abbreviation**
HDR: High‐Dose‐Rate
DIL: Dominant Intraprostatic Lesion
CT: Computed Tomography
MRI: Magnetic Resonance Imaging
TRUS: Transrectal‐Ultrasound
RR: Rigid Registration
CB: Contour‐Based Registration
pfRR: Predicted Fusion Followed by Rigid Registration
pfCB: Predicted Fusion Followed by Contour‐Based Registration
MDA: Mean Distance to Agreement

## 1 INTRODUCTION

High‐dose‐rate (HDR) prostate brachytherapy is an established treatment technique, in combination with external beam radiotherapy, for intermediate‐ and high‐risk prostate cancer.^1^, ^2^ The current approach to prostate cancer radiotherapy involves the irradiation of the entire gland. In recent years, interest has been mounting in treating the prostate using a focal therapy approach. This can involve either escalating the dominant intraprostatic lesions (DILs) of disease to a higher boost dose while maintaining the dose to the entire prostate, treating half of the prostate (termed “hemigland” treatment), or exclusively treating the DIL(s). A summary of select number of focal brachytherapy studies is provided in Table 1.

**Table 1:** Literature summary for select focal brachytherapy studies.

Study	Focal study design	Image fusion strategy	Results
Dosimetry investigations			
Zaider et al. (2000)^22^	LDR brachytherapy Case study Whole prostate treated with boost to DIL	Geometric rigid fusion between MRSI and TRUS	Feasible to escalate dose using developed optimization system
Pouliot et al. (2004)^23^	HDR brachytherapy Whole prostate treated with boost to DIL	DIL manually drawn on CT or MRI planning scan using MRI/MRSI	Feasible to escalate dose to the DIL to 120% of the prescription dose without compromising OAR dose constraints
Todor et al. (2011)^24^	LDR brachytherapy Whole prostate treated with boost to DIL	Manual rigid registration	Explored use of two different isotopes to optimize the DIL boost (“could be viewed as an intermediate solution between whole‐gland irradiation and focal treatment”)
Mason et al. (2014a)^25^	HDR brachytherapy Whole prostate treated with boost to DIL	Manual rigid registration	Feasible to escalate dose to the DIL to 120%‐150% of the prescription dose
Mason et al. (2014b)^26^	HDR brachytherapy Ultrafocal treatment (i.e., only the DIL with margin was treated); also investigated hemigland treatment	Not required	Feasible to escalate dose to the DIL while reducing OAR dose; focal plans were more susceptible to source positioning errors than whole prostate
Dankulchai et al. (2014)^27^	HDR brachytherapy Whole prostate treated with boost to DIL	Not required	Feasible to dose escalate the DIL to 110% of the prescription dose in the majority of patients; improved when needles are spaced closer together
Hosni et al. (2017)^28^	HDR brachytherapy Ultrafocal treatment (i.e., only the DIL with margin was treated)	Deformable image registration	Feasible to dose escalate the DIL with margin in either one or two implants for most patients
Clinical investigations			
DiBiase et al. (2002)^29^	LDR brachytherapy (^125^I) Low‐risk patients Whole prostate treated with boost to DIL (135% of the prescription dose)	Target from MRSI geometrically described and then drawn on TRUS	Feasible to use MRSI to guide a focal dose escalation; low rectal and urethral morbidity
Cosset et al. (2013)^30^	LDR brachytherapy (^125^I) Low‐risk patients Ultrafocal treatment (i.e., only the DIL with margin was treated)	DIL drawn on ultrasound planning scan using biopsy sites and MRI	Reduction in urinary toxicity compared to previous whole‐gland brachytherapy cohorts at 6 months
Barret et al. (2013)^31^	LDR brachytherapy (^125^I), among other forms of focal therapy Low‐risk patients Trial included 106 patients treated with focal therapy using high‐intensity focal therapy, brachytherapy, cryotherapy, or photodynamic therapy	NR	No grade ≥2 complications in the brachytherapy arm
Crook et al., 2014^32^	HDR brachytherapy Intermediate‐ and high‐risk patients Whole prostate treated with boost to DIL (125% of the prescription dose)	Manual rigid registration	No clinical outcomes reported; feasible to dose escalate the DIL
Vigneault et al. (2016)^33^	HDR brachytherapy Intermediate‐risk patients Whole prostate treated with boost to DIL (120% of the prescription dose)	NR	5 year biochemical failure‐free survival: 94.7% Acceptable acute and late gastrourinary and gastrointestinal toxicities
Gomez‐Iturriaga et al. (2016)^34^	HDR brachytherapy Intermediate‐ and high‐risk patients Whole prostate treated with boost to DIL (125% of the prescription dose)	Rigid registration from pre‐treatment MRI to secondary MRI, taken with rectal cylinder inserted on day of implant, then to intraoperative TRUS	No patients developed grade ≥3 toxicity at 18 month median follow‐up
Maenhout et al. (2018)^35^	HDR brachytherapy Mostly low‐ and intermediate‐risk patients Ultrafocal treatment (i.e., only the DIL with margin was treated)	N/A (MRI‐guided)	Low toxicity High recurrence rate with 2 year median follow‐up
Tissaverasinghe et al. (2019)^36^	LDR & HDR brachytherapy (Phase II trial) Mostly intermediate risk Whole prostate treated with boost to DIL (125% of the prescription dose for HDR brachytherapy, 150% of the prescription dose for LDR brachytherapy)	Contour‐based rigid registration (pre‐treatment TRUS to MRI), then to intraoperative TRUS	Dose escalation was feasible with both LDR and HDR brachytherapy (when target close to organs‐at‐risk, HDR may perform better)

Studies reporting on dose escalation/targeting of the entire peripheral zone or hemigland treatments are not included here due to the differing target definition requirements that may not require image fusion or margin strategies. Studies restricted to focal salvage therapy are also not reported here due to the potential for different dosimetric constraints.

Abbreviations: DIL, dominant intraprostatic lesion; HDR, high‐dose rate; LDR, low‐dose rate; MRI, magnetic resonance imaging; MRSI, magnetic resonance spectroscopic imaging; NR, not reported; OAR, organ at risk; TRUS, transrectal ultrasound.

HDR prostate brachytherapy relies heavily on imaging infrastructure and can be delivered using a variety of imaging workflows including integration with computed tomography (CT), magnetic resonance imaging (MRI), and transrectal ultrasound (TRUS). TRUS guidance in HDR brachytherapy has been widely used,^3^ due largely to its cost‐effectiveness and availability. Unfortunately, however, soft‐tissue resolution on TRUS imaging is poor, creating challenges in resolving intraprostatic features. MRI, in contrast, excels at soft tissue contrast and has been increasingly incorporated into radiotherapy practices to aid with segmentation of both cancerous targets and organs at risk. Historically, the fusion between MRI and TRUS images has only been possible using cognitive registration; however, software‐based tools are gradually being introduced into brachytherapy.^4^

While several MRI‐TRUS fusion tools have been described in literature, both for targeted prostate biopsy and for brachytherapy applications,^5^, ^6^, ^7^, ^8^, ^9^, ^10^, ^11^, ^12^, ^13^, ^14^, ^15^ it is important for centers to independently assess registration methodologies for use in their own clinical workflow. The registration details along with major results of these MRI‐TRUS studies are summarized in Table 2, along with these details in relation to the major results from this study. Differences in MRI specifics, including magnetic field strength and use of endorectal coils, and HDR brachytherapy planning strategies can result in changes to the registration accuracy and necessary precision. This study reports the first commercial solution to fuse the MRI and the TRUS images specifically for HDR brachytherapy available in the MIM treatment planning system (MIM Software Inc., Cleveland OH).

**Table 2:** Selected literature summary for studies assessing MRI‐TRUS registration methodologies and algorithms for use in brachytherapy.

Study	Registration details from study	Registration methodology	Results
Current study	Twenty patients. RR and DIR MIM registrations assessed with and without use of MIM's predictive fusion tool. Additional preliminary assessment of landmark‐based registration for subset of five patients.	Rigid^a^	Dice: 0.80 ± 0.05 MDA: 2.1 ± 0.8 mm
		Deformable^a^	Dice: 0.93 ± 0.02 MDA: 0.60 ± 0.08 mm
Shaaer et al. (2018)^9^	Ten patients in study. Cognitive‐based contouring of DIL TRUS images using MRI as reference. In‐house registration technique—combo of RR and DIR (in‐house and *B*‐spline)	Rigid	Dice (DIL): 0.65 ± 0.20 MDA: 1.71 ± 0.80 mm
		Deformable (in‐house)	Dice (DIL): 0.80 ± 0.13 MDA: 1.30 ± 0.53 mm
		Deformable (*B*‐spline)	Dice (DIL): 0.51 ± 0.30 MDA: 3.10 ± 2.00 mm
Poulin et al. (2018)^6^	Fifteen patients in study. Registration module incorporated into open‐source 3D slicer platform. RR and DIR investigated.	Rigid	Dice: 0.87 ± 0.05 Hausdorff (95%): 4.2 ± 1.0 mm TRE: 3.5 ± 3.2 mm
		Deformable	Dice: 0.93 ± 0.01 Hausdorff (95%): 2.2 ± 0.3 mm TRE: 2.3 ± 1.1 mm
Mayer et al. (2016)^14^	Ten patients. Six with landmarks (e.g., brachytherapy seeds) and four with EBRT implanted fiducials visible in both modalities. DIR via in‐house *B*‐spline approach for the fiducials and landmarks.	Deformasble	TRE: 2.6 ± 1.3 mm
Fedorov et al. (2015)^8^	Eleven patients. Open‐source, two DIR methods investigated—DIR of prostate segmentation maps with *B*‐spline regularization and a finite element‐based DIR of segmentation surfaces in presence of partial DIR data.	Deformable (B‐spline)	TRE: 3.8 ± 1.8 mm
		Deformable (finite‐element)	TRE: 3.5 ± 1.7 mm
Hu et al. (2012)^15^	Eight patients. DIR approach using an in‐house novel “model‐to‐image” approach for prostate gland. Anatomical landmarks used to quantify registration accuracy. Final TRE presented for target registration after performing 100 MR‐TRUS registrations for each patient	Rigid	TRE: 5.1 mm (95% CI: 12.1 mm)
		Deformable	TRE: 2.4 mm (95% CI: 6.2 mm)
Reynier et al. (2004)^13^	Eleven patients as well as results for a phantom study presented. PROCUR system used for registration ‐ RR and DIR (elastic). Used for LDR permanent implant patients.	Rigid	Residual distance of surface points: 1.4 ± 0.2 mm
		Deformable	Residual distance of surface points: 1.1 ± 0.5 mm

Abbreviations: CI, confidence interval; DIL, dominant intraprostatic lesion; DIR, deformable image registration; MDA, mean distance to agreement; NR, not reported; RR, rigid registration; TRE, target registration error.

^a^ Results presented for rigid and deformable registrations without Predictive Fusion applied.

In this work, we evaluate two multimodality image registration methodologies within the MIM treatment planning system to fuse the MRI to the TRUS images: rigid registration (RR) and contour‐based (CB) deformable image registration. These are assessed in combination with the Predictive Fusion workflow specific to the MIM treatment planning system, which allows the user to identify the expected location of the TRUS probe during brachytherapy on the MRI and reorient the slices of the MRI perpendicular to the probe angle in an effort to improve the fusion by accounting for patient positioning differences.

Additionally, this study provides a preliminary investigation into the application of this workflow in the context of DIL‐based foci therapy. Specifically, exploration into intraprostatic landmark‐based approach was investigated using patient‐specific landmarks.

## 2 METHODS

### 2.A Patient characteristics

Twenty consecutive patients treated with HDR prostate brachytherapy at the Tom Baker Cancer Centre, Calgary, AB, were included in this retrospective evaluation. All patients received this treatment as a component of their combined modality treatment for intermediate‐ or high‐risk prostate cancer, receiving 15 Gy in one fraction via HDR prostate brachytherapy and 46 Gy in 23 fractions via external beam radiotherapy. Intermediate‐ and high‐risk prostate cancers were defined as Gleason 7+, PSA 5+, with a required software‐guided, targeted biopsy with UroNav system (Invivo, Gainesville, FL) using mpMRI to confirm Gleason score. A selection of relevant patient characteristics is shown in Table 3.

**Table 3:** Characteristics for patients (*N* = 20) included in this retrospective study.

Parameter	Mean ± standard deviation *or N* (%)
Age (years)	66 ± 4
Prostate volume (cm^3^)	
TRUS	31 ± 15
MRI	35 ± 20
Time between MRI and HDR brachytherapy (days)	45 ± 45
External beam radiotherapy prior to HDR brachytherapy	11 (60%)

### 2.B Pre‐treatment magnetic resonance imaging

**Fig. 1:**
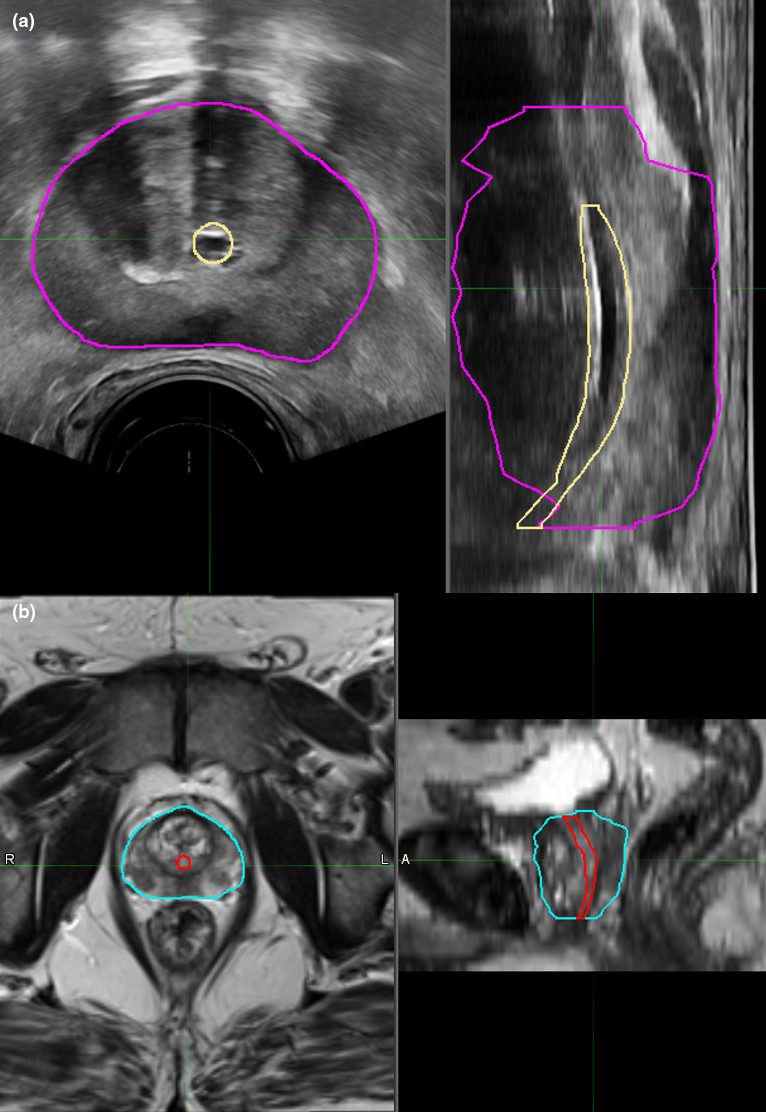
Sample axial and sagittal TRUS (a) and MRI (b) images from this study. The prostate is contoured in purple and cyan and the urethra is contoured in yellow and red on the TRUS and MRI, respectively.

All patients underwent a multiparametric MRI scan in advance of HDR prostate brachytherapy. Multiparametric sequences included T1‐ and T2‐weighted scans, diffusion‐weighted imaging, and gadolinium contrast‐enhanced sequences performed using a 3‐T magnet. No endorectal coil was used. The MRI was resampled in the MIM treatment planning system to 1‐mm isotropic resolution to match the superior‐inferior resolution of the 3D TRUS scan. The prostate was delineated by a radiation oncologist on the resampled T2‐weighted image. An example of an MRI scan from one of the study patients is shown in Figure 1.

### 2.C Clinical brachytherapy process

The HDR prostate brachytherapy process at the Tom Baker Cancer Centre, Calgary, AB, follows a TRUS‐guided, intraoperative‐planned approach. The implant is performed with the patient under spinal anesthesia in an unshielded operating room. The patient is positioned supine in the lithotomy position with a TRUS probe inserted. A pre‐implant 3D TRUS image is manually acquired at 1‐mm superior–inferior spacing for catheter location planning; an example of such an image is shown in Figure 1. For use in this study, the prostate was retrospectively contoured by a radiation oncologist on this image. Catheters are subsequently inserted transperineally. A 3D TRUS image is reacquired following completion of catheter insertion to use for planning. Following planning, the patient is transferred to a shielded HDR vault adjacent to the operating room for treatment delivery.

HDR brachytherapy is exclusively delivered as a component of a combined modality regimen at our center at present. All patients undergo one fraction of HDR brachytherapy (prescription dose of 15 Gy) and a 23‐fraction course of external beam radiotherapy (prescription dose of 46 Gy). The two treatment components may be delivered in either order. Hormonal therapy may also be offered.

### 2.D Image registration and evaluation

**Fig. 2:**
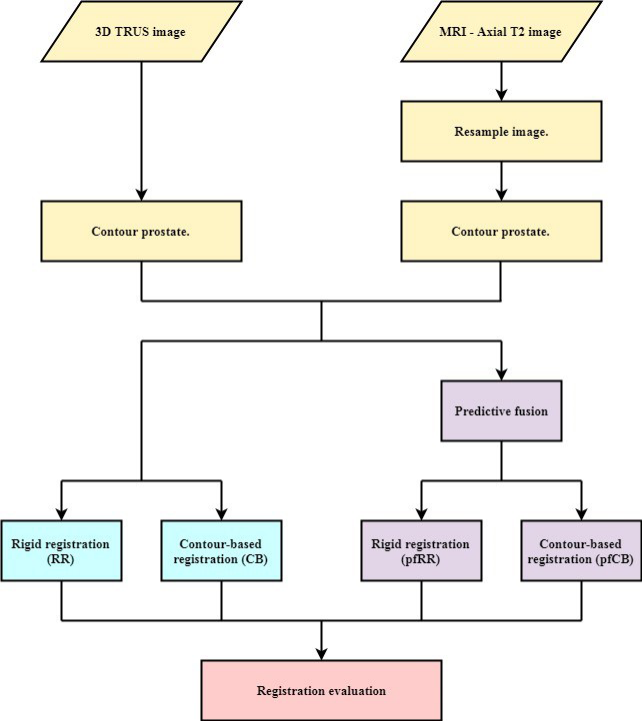
Image registration and evaluation workflow.

For this retrospective study, the pre‐brachytherapy MRI and TRUS acquired during brachytherapy were used to investigate and evaluate the quality of MIM‐based image registration for twenty patients. Both MRI and US datasets had the prostate, urethra, and rectal wall contoured by the radiation oncologist. The image registration workflow using MIM is shown in Figure 2. Two registration techniques for the 20 multimodality (MRI and TRUS) imaging datasets were investigated: RR and CB deformable registration. Each was performed either “as is” or preceded by the MIM Predictive Fusion workflow to assess its impact. Thus, in total, four variations of registration strategies were assessed for each of the 20 patient datasets: RR, CB registration, Predictive Fusion followed by RR (pfRR), and Predictive Fusion followed by CB registration (pfCB).

During RRs, the 3D MRI dataset was manually fused with the 3D TRUS imageset. This was performed by aligning the prostate and the anterior rectal wall between the two images. The RR only utilized translations, not rotations. Rotations were excluded due to literature suggesting that the inclusion of a rotation for the registration of the prostate may degrade the quality of the registration^9^; further, the registrations performed with Predictive Fusion isolated the rotation about the cranial–caudal axis, hypothesized to be the largest axis of rotation. The CB deformable registration used this manual RR as a starting point and subsequently deformed the MRI to the TRUS image using the prostate contours on each image.

**Fig. 3:**
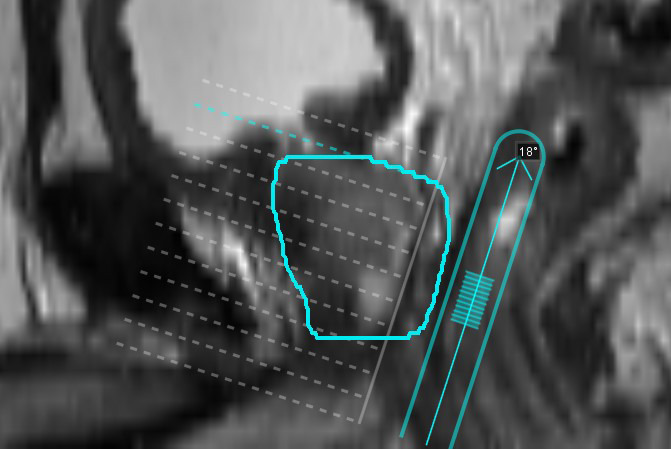
Predictive Fusion workflow in the MIM treatment planning system. The prostate contour on this sagittal slice is shown in cyan. The TRUS probe is positioned in its expected location in the rectum, as shown (angled at 18°), and the MRI is resliced perpendicular to this probe orientation to attempt to match patient positioning for the TRUS scan and brachytherapy procedure.

The Predictive Fusion workflow within MIM allows the user to position and orient the TRUS probe on the MRI scan. The software then produces a “resliced” MRI that has been reoriented perpendicular to the TRUS probe. This process is shown in Figure 3. No deformation is introduced in this fusion process. After performing the predictive fusion, a RR or CB registration was performed as previously described.

To evaluate the geometric quality of the four variations of registration strategies for each patient, a distance‐based metric (mean distance to agreement [MDA]) and a volume‐based metric (Dice similarity coefficient) were used, as per AAPM TG‐132 recommendations.^16^ These metrics compared the expert‐delineated prostate contour on the TRUS image with the contour registered from the MRI. The impact of including the Predictive Fusion workflow was statistically quantified using the Wilcoxon signed‐rank test. As all registrations relied upon a manual RR as a starting point, all registrations were repeated three times to assess variability. The median of the metrics obtained in the three iterations was used for statistical analysis. For the registrations that included Predictive Fusion (pfRR and pfCB), the probe was manually repositioned each time without using previous iterations to assess the consistency of defining this angle. All deformable registrations were also qualitatively assessed; the deformed MRI was reviewed for non‐physically feasible deformations. The volume of the deformed MRI contours and the original TRUS contours were compared. All statistical analysis was performed in Matlab R2016a (The Mathworks Inc., Natick MA).

### 2.E Preliminary investigation of landmark‐based registrations

Although this study is primarily focused on assessing and quantifying MIM registration workflow in the context of HDR prostate brachytherapy via the RO‐delineated prostate contour, a preliminary investigation into intraprostatic landmark‐based registration between MRI and TRUS imagesets was also explored. For a subset of five of the 20 retrospective study patients, the patient‐specific landmarks included: nodule, cyst, fluid pocket, and calcification. These five patients were chosen such that they had one of the aforementioned landmarks anatomic landmarks visible on both MRI and TRUS imagesets. The same registration strategies were investigated for these landmarks, and the DICE and MDA metrics were calculated to assess the performance.

## 3 RESULTS

### 3.A Image registration evaluation

**Fig. 4:**
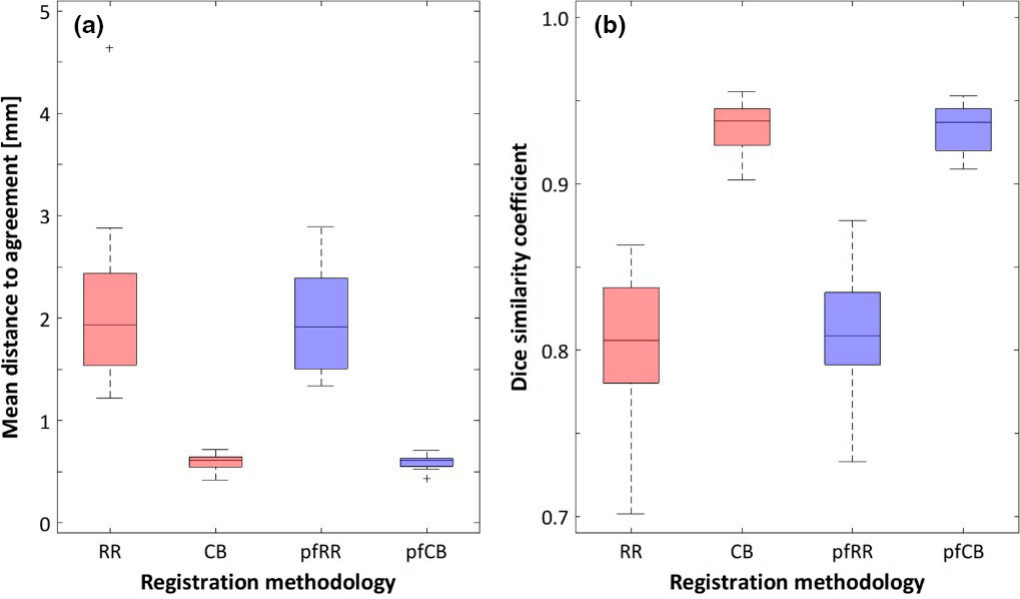
Geometric comparisons for prostate contours (expert‐drawn contours on TRUS and registered MRI contours) using (a) mean distance to agreement and (b) the Dice similarity coefficient. Smaller mean distance to agreement and larger Dice similarity coefficient indicate better geometric agreement between contours. In these boxplot figures, the line denotes the median of the distribution (N=20 patients) while the box denotes the interquartile range (IQR; 25th to 75th percentile). Outliers, denoted with ‘+’, are defined as points >1.5×IQR below or above the 25th or 75th percentile, respectively. The whiskers extend to the most extreme points, excluding outliers.

The four registrations investigated are compared in Figure 4 using the MDA and the Dice similarity coefficient. MDA was 2.1 ± 0.8 mm, 0.60 ± 0.08 mm, 2.0 ± 0.5 mm, and 0.59 ± 0.06 mm for RR, CB, pfRR, and pfCB, respectively. Dice similarity coefficients were 0.80 ± 0.05, 0.93 ± 0.02, 0.81 ± 0.03, and 0.93 ± 0.01 for RR, CB, pfRR, and pfCB, respectively. The inclusion of the Predictive Fusion workflow did not lead to a significant improvement in MDA or Dice similarity coefficient for either RR or CB registration. For both metrics, CB registration performed significantly better than RR both when Predictive Fusion was included (i.e., *pfCB* compared to *pfRR*) and when it was not included (i.e., *CB* compared to *RR*).

When positioning the TRUS probe on the MRI for the Predictive Fusion workflow, the angle selected was an average ± standard deviation 12 ± 5° from the cranial–caudal axis (head‐down, as shown in Figure 3). The intrapatient variation was 3 ± 3° among the three independent angle selections.

**Fig. 5:**
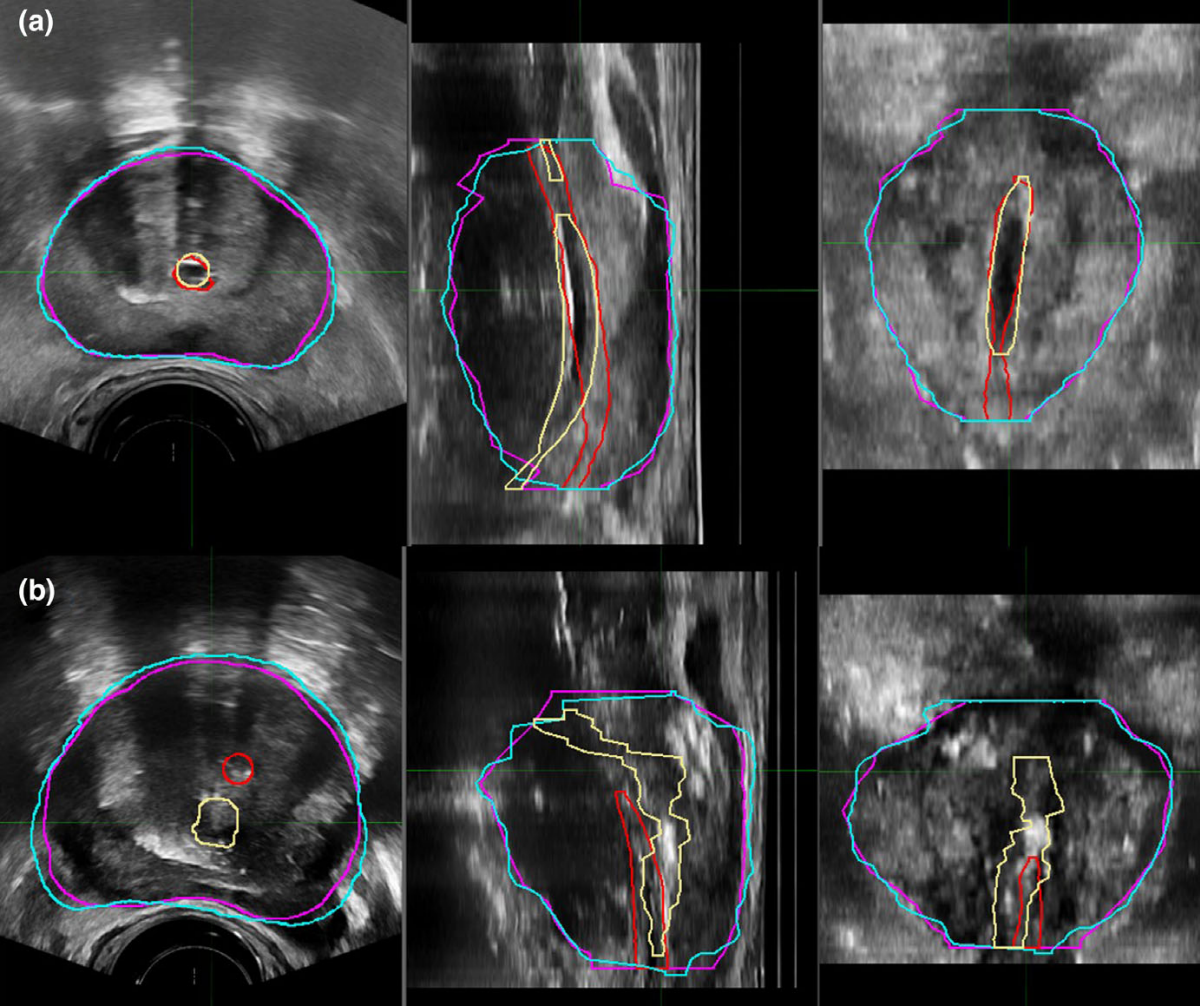
Example of a good (a) and a poor (b) contour‐based deformable image registration. In both panels, the TRUS contours for the prostate and urethra are shown in purple and red, respectively. The deformed MRI contours for the prostate and urethra are shown in cyan and yellow, respectively.

The MRI prostate volumes were average ± standard deviation 6 ± 17% larger than the TRUS prostate volumes. The deformed MRI contours following CB registration, however, exhibited far greater consistency with the TRUS volumes, with volume differences of average ± standard deviation −1 ± 4% for both *CB* and *pfCB*. All deformed MRIs were qualitatively assessed for any physically infeasible deformations that may not be captured in the assessment of geometric indices alone; no such abnormalities were identified. When qualitatively assessing the deformed MRI contours, inconsistencies existed most frequently in the base region, with some non‐contiguities in deformed contours observed. An example of both a good and a poor CB deformable registration is shown in Figure 5. Similar issues were encountered in the apex. Deviations in the deformed contours also existed in patients where the prostate contour on TRUS displayed a prominent curvature around the probe. In the context of DIL‐based registrations, DILs located in these regions (e.g., the base, apex, or posterior‐lateral edges of the prostate) should be particularly scrutinized on their transfer to the TRUS image.

### 3.B Preliminary anatomical landmark‐based registration

All landmark‐based registrations for the five patients generated a MDA of less than 2 mm when comparing MRI contours propagated using CB deformable image registration to their TRUS‐contoured counterparts. The urethra and proximal seminal vesicle anatomical landmarks, however, performed poorly with MDA values for some patients reaching maximum values of 4.1 and 6.3 mm, respectively.

## 4 DISCUSSION

In recent years, the use of HDR brachytherapy as a component of the treatment pathway for prostate cancer, particularly for intermediate‐ and high‐risk patients, has increased.^2^ In conjunction with this, there has been growing interest in focal therapy for prostate cancer in both primary and boost settings; HDR brachytherapy is an ideal candidate to achieve this targeting due to its steep dose gradients and high precision abilities. In TRUS‐guided HDR brachytherapy procedures, however, substantial uncertainty is present in administering focal therapy due to the lack of intraprostatic soft tissue resolution. Similarly, in TRUS‐guided biopsies, where cores of prostate tissue are removed for pathological assessment, visualization of lesions of disease during the procedure is not possible. The radiology and urology communities have developed substantial expertise in TRUS‐MRI fusion (both rigid and elastic applications), starting from the early 2000s,^7^ to permit targeted biopsies, with the radiation oncology community now seeking to follow a similar path. A summary of studies investigating TRUS‐MRI registration for application in brachytherapy can be found in Table 2. The large number of studies reflects the diversity in study design and infrastructure, including differences in magnetic field strength, use of endorectal coils, registration algorithm availability, and application (for example, biopsy, whole‐gland brachytherapy, focal brachytherapy, etc.). In turn, this reflects the importance of each investigation and the selected implementation of TRUS‐MRI registration. Consistent with the recommendations of TG‐132,^16^ it is important that each center quantitatively validate their own process for image registration to ensure accurate and safe implementation. Validation of intermodality prostate fusion has been performed following various geometric approaches. While some studies have used the urethral entry and exit into the prostate as a landmark for validation, we opted not to in our study due to the observed instability of this positioning intraoperatively during the HDR brachytherapy process, compounded by differences in catheterization between images and other positioning differences.

HDR prostate brachytherapy is commonly performed as an intraoperative procedure. While MRI‐based workflows are becoming more common,^19^ the high cost and availability of the equipment still limit their availability. Ultrasound, conversely, is cost‐effective and widely available and requires minimal space and specialized infrastructure.^19^ It has been used extensively in prostate brachytherapy procedures yielding practitioner comfort and experience. MRI‐TRUS fusion strategies will allow the use of MRI soft‐tissue information in a TRUS‐guided procedure, improving the ability for centers to implement targeted strategies while maintaining their TRUS‐guided procedure. In this study, MRIs were obtained with wide variability in timing prior to brachytherapy (see Table 3); these images were required for patients' standard clinical care, not specifically for use in this study. The ability of the image fusion algorithm to perform with high consistency on these images is further indication of the strength of the MIM CB deformable image registration algorithm. Further, in this study, all contouring was done retrospectively on static images. In a clinical implementation, it is expected that the MRI would be contoured in advance of the intraoperative procedure while the fusion would be performed in the operating room. This study used a static, 3D TRUS image for the fusion; however, in clinical practice, it is possible that this fusion could be done on a live image. The accuracy of this would need to be quantified and may offer a scenario where Predictive Fusion is better suited. Varying processes for the clinical implementation of this registration may be considered due to anatomical changes to the prostate introduced by catheter insertion.^20^ In this study, the MRI was fused with the TRUS performed prior to catheter insertion; recent literature has suggested that efficiency may be gained by instead registering with the TRUS image taken after catheter insertion (that is, the image used for planning).^6^

This study relied on geometric indices to quantify the quality of the MRI‐TRUS registration. Such geometric indices are commonly used in radiotherapy studies as surrogates for the determination of the adequacy of the dosimetry (i.e., if it is consistent when calculated on either contour). As a community, we still seek definitive dosimetric indices for HDR prostate brachytherapy; varying prescription doses (including adequate single‐fraction monotherapy doses,^21^ for example) and fractionations remain under investigation.

We elected to use the prostate contours in this study for registration as these are well visualized on both MRI and TRUS imaging modalities. Implementation in our clinical process would thus involve contouring of the prostate intraoperatively. Exceptional agreement of the prostate contour registration metrics, MDA and DICE (Figure 4), lends confidence to this process; as uncertainties were identified in the base and apex regions, however, critical dosimetric assessment at those locations (particularly in the case of disease located in those locations) would be warranted in an intraoperative setting.

Rotation of the coordinate system when comparing an MRI, which is obtained with the legs down to allow the patient to fit inside the imager bore, and the TRUS, which is obtained in the lithotomy position, has been previously reported in the literature,^11^, ^15^ motivating solutions such as the Predictive Fusion option offered by MIM. It has, however, also been reported that including rotation in prostate RRs may degrade registration quality.^9^ In this study, the CB deformable registration was performed after three separate manual RRs, given the potential for variation in the manual alignment. The results for the deformed prostate in each of the three iterations were remarkably similar, demonstrating the strength of the performance of these deformable registration algorithms and the potential for a decreased need to focus on prostate rotations. This is further demonstrated by the negligible difference in both MDA and DICE coefficients when comparing the RR and CB registrations with and without the Predictive Fusion applied (Figure 4). This suggests that both RR and CB registrations in MIM are robust and able to accommodate the aforementioned changes in anatomy between the MRI and TRUS imagesets due to leg position and US probe‐in vs. probe‐out, without need of applying the additional Predictive Fusion option.

A gold standard for the registration of the DIL does not currently exist. While some studies have compared cognitive contouring of the DIL with that obtained by the registration, this is not a true reference. The presence of implanted fiducial markers could provide a stable surrogate for the DIL that is well‐visualized in all imaging modalities. Due to the retrospective nature of this study, the patients imagesets did not contain implanted fiducial markers that could be used to validate DIL registration between the MRI and TRUS imagesets.^17^, ^18^ Some alternative, although less accurate, approaches for validation of DIL registration include assessing the accuracy of the propagation of intraprostatic anatomical landmarks using deformable‐image‐registration‐generated MRI contours to TRUS contours as a surrogate for the DIL. These landmarks include the urethra, proximal seminal vesicles, as well as the patient‐specific landmarks such as nodules, cysts, fluid pockets, and calcifications. Additionally, comparison of cognitive contouring of the DIL on TRUS images to deformable‐image‐registration‐propagated DIL contours from the MRI can be a means of checking for gross errors in the registrations.^9^

This study also provides a preliminary investigation into the performance of this MIM‐based workflow for applications that may have implications for DIL‐based foci HDR therapy, as well as recommendations to improve the accuracy of implementation and thus reducing the required safety margin to ensure treatment accuracy in foci therapy.

The intraprostatic landmark registration approach showed promise from the preliminary investigation on five of the 20 retrospective patients. All generated an MDA of less than 2 mm when comparing MRI contours propagated using CB DIR to their TRUS‐contoured counterparts. The urethra and proximal seminal vesicle anatomical landmarks, however, performed poorly with MDA values for some patients reaching maximum values of 4.1 and 6.3 mm, respectively. This may be attributed to the noticeably different imaging conditions between the MRI and TRUS, with the lack of endorectal coils and catheterization in MRI most likely having a major impact on urethra positioning between the two image datasets. Future studies should look to use implanted fiducials to ensure minimal registration error for the DIL. This reduced registration error could allow for a reduced safety margin to the DIL, which is typically added to account for planning and treatment uncertainties. This should result in reduced dose to surrounding healthy tissue while still ensuring appropriate coverage of the DIL. Future studies will also require assessment of the dosimetric uncertainties imparted by any geometric deviations. While geometric indices have often been used as the standard for validation of image registration quality, dosimetric accuracy must be investigated to provide clinical context.

The results of this study for MRI‐TRUS registration in the context of brachytherapy show comparable, and in some cases, improved results compared to previous studies (Table 2). Additionally, this was achieved with previously validated registration algorithms available in a well‐known commercial platform, MIM, while other studies implemented algorithms created in‐house and open‐source which have not necessarily gone through the same rigorous regulatory testing as a commercial software. With MIM offering a variety of other tools (e.g., contouring) that could be used in addition to the MRI‐TRUS registration investigated in this study, MIM could be a valuable platform, streamlining the workflow in focal therapy for prostate cancer.

Focal therapy for prostate cancer is an area of active research across a number of medical communities. It is hypothesized that this treatment strategy can lead to reduced patient side effects while maintaining disease control. A summary of reported focal therapy investigations is provided in Table 1; several groups have undertaken dosimetric and clinical studies to determine the optimal delivery of focal therapy via brachytherapy. Extensive variability exists in these studies; and the interpretation of the results is compounded by uncertainties in prescription dose and margin utility.

## 5 CONCLUSIONS

In this study, we have reported the first commercial brachytherapy solution for TRUS‐MRI fusion in the MIM treatment planning system. CB deformable registration yielded superior geometric results compared to RR when considering the external prostate contour. Utilizing the predictive fusion tool yielded similar results for the geometric indices, MDA and DICE, for both rigid and CB DIR in absence of the tool and was not seen as mandatory for the MRI‐TRUS fusion workflow. This MRI‐TRUS based workflow looks promising for application in HDR foci‐therapy for prostate brachytherapy.

## CONFLICTS OF INTEREST

The authors have no relevant conflicts of interest to disclose.

## AUTHOR CONTRIBUTIONS

Philip McGeachy: design study, data analysis and workflow, author of manuscript, reviewed manuscript and respond to reviewers. Elizabeth Watt: design study, data analysis, co‐author of manuscript, reviewed manuscript. Siraj Husain: data acquisition (contouring for prostate patients on MRI and TRUS), reviewed manuscript. Kevin Martell: data acquisition (contouring for prostate patients on MRI and TRUS), reviewed manuscript. Pedro Martinez: data analysis (performed several registrations techniques for dataset), reviewed manuscript. Summit Sawhey: data acquisition (contouring for prostate patients on MRI and TRUS), reviewed manuscript. Kundan Thind: design study, data analysis, review and edit manuscript.

## Data Availability

The data that support the findings of this study are available on request from the corresponding author. The data are not publicly available due to privacy or ethical restrictions.
